# High-Resolution Ultrasound Evaluation of Structural Remodeling After Fat Grafting in Irradiated Chest Wall Tissues

**DOI:** 10.3390/diagnostics16040511

**Published:** 2026-02-08

**Authors:** Razvan-George Bogdan, Mara Nicolau, Alina Helgiu, Anca-Maria Campean, Zorin Petrisor Crainiceanu

**Affiliations:** 1 Doctoral School, “Victor Babeș” University of Medicine and Pharmacy Timișoara, 300041 Timișoara, Romania; razvan.bogdan@umft.ro; 2 County Clinical Emergency Hospital “Pius Brînzeu”, 300723 Timișoara, Romania; crainiceanu.zorin@umft.ro; 3 Faculty of Medicine, “Lucian Blaga” University of Sibiu, 550024 Sibiu, Romania; mara.nicolau16@gmail.com; 4 County Clinical Emergency Hospital of Sibiu, 550245 Sibiu, Romania; 5 Department of Microscopic Morphology/Histology, Victor Babes University of Medicine and Pharmacy Timisoara, 300041 Timisoara, Romania; 6 Center of Expertise for Rare Vascular Disease in Children, Emergency Hospital for Children Louis Turcanu, 300011 Timisoara, Romania; 7 Center of Genomic Medicine, Victor Babeș University of Medicine and Pharmacy, 300041 Timisoara, Romania; 8 Research Center for Pharmaco-Toxicological Evaluation, Victor Babes University of Medicine and Pharmacy, 300041 Timisoara, Romania; 9 Plastic Surgery Department, “Victor Babeș” University of Medicine and Pharmacy, 300041 Timișoara, Romania

**Keywords:** autologous fat grafting, ultrasound, irradiated tissue, breast reconstruction, fibrosis, tissue remodeling

## Abstract

**Background:** Autologous fat grafting is increasingly used in irradiated postmastectomy tissues, but objective imaging-based data describing structural remodeling remain limited. **Objective:** This pilot study aimed to evaluate ultrasound detectable structural changes following autologous fat grafting in irradiated postmastectomy chest wall tissues. **Methods:** This prospective pilot study included five female patients with prior radical mastectomy and adjuvant chest wall radiotherapy who underwent a single-session of autologous fat grafting. High-resolution ultrasound was performed preoperatively and at 3–5 months postoperatively using a 12 MHz linear probe. Parameters evaluated included hypodermal thickness, echogenicity (hyperechoic versus hypoechoic patterns), fascial definition, and fibrotic patterns. **Results:** All patients demonstrated a consistent postoperative increase in hypodermal thickness. Preoperative compact, hyperechogenic architecture transitioned to heterogeneous hypoechogenic patterns suggestive of viable adipose tissue integration consistent with viable adipose tissue. Fascial planes became more clearly defined in four patients. No necrosis, oil cysts, or fluid collections were detected. **Conclusions:** In this pilot cohort, ultrasound detected consistent postoperative changes in hypodermal thickness, echogenicity, and fascial definition following autologous fat grafting. These findings support the feasibility of ultrasound for the non-invasive assessment of post-radiotherapy structural tissue changes.

## 1. Introduction

The most prevalent disease in women globally is still breast cancer, and during the past few decades, improvements in oncologic treatment, such as surgery, chemotherapy, and radiation, have greatly increased the survival rates [1]. Among these, adjuvant radiation is essential for lowering locoregional recurrence, although it frequently has detrimental side effects on the epidermis and subcutaneous layers of the chest wall [2]. Dermal and hypodermal fibrosis, subcutaneous atrophy, reduced vascularity, and chronic inflammation are common in irradiated tissues, and they can significantly affect postmastectomy patients’ quality of life and reconstructive results. Although radiotherapy is essential for improving the oncologic results of breast cancer, it frequently causes long-term harm to the skin and subcutaneous tissues. The effectiveness of secondary breast reconstruction, especially with implants, is severely limited by these alterations, which include fibrosis, microvascular impairment, dermal atrophy, and reduced tissue compliance [3,4].

Radiation-induced histological and biomechanical alterations produce stiff, fibrotic, and poorly perfused tissues that are frequently inappropriate for flap-based restorations or traditional prosthetics [5]. In addition to reducing the number of reconstructive choices available, these aftereffects also exacerbate chronic complaints such as pain, pruritus, scar contracture, and dissatisfaction with appearance [6]. In this regard, autologous fat grafting (AFG), also referred to as lipofilling, has been increasingly used as an adjunctive reconstructive technique that is being used more and more in oncologic breast surgery. Fat grafting was first suggested as a contour correction technique, but is now known to be associated with improvements in tissue quality in irradiated areas [7].

Autologous fat grafting has been used as an adjunctive reconstructive approach in irradiated tissues. In addition to its volumetric advantages, lipofilling has been demonstrated to enhance skin quality, reduce discomfort following mastectomy, and restore fibrotic tissue’s pliability [8,9]. The presence of adipose-derived stromal/stem cells (ADSCs), which regulate inflammation, lower fibrotic activity, and encourage angiogenesis and extracellular matrix remodeling (ECM), has been associated with these effects in previous experimental and clinical studies [10,11]. These mechanisms have been described in histological and experimental studies. After fat grafting, Rigotti et al. showed neovascularization and structural repair in irradiated tissue [11]. Clinical studies by Caviggioli et al. revealed reductions in pain and scar pliability, confirming the fat grafting’s wider therapeutic applicability in this patient group [8].

A standardized, non-invasive technique for assessing graft integration and tissue remodeling is still missing, despite the fact that fat grafting is widely used. Subcutaneous echotexture, thickness, and fascial plane reappearance may all be evaluated using ultrasound, a real-time, affordable, and repeatable imaging method [3]. Particularly in reconstructive contexts including earlier irradiation, recent work emphasizes the necessity of standardized imaging techniques to guarantee repeatability and enable interstudy comparisons [12,13]. Recent clinical data have demonstrated that autologous fat grafting can lead to a measurable increase in mastectomy flap thickness, supporting the relevance of objective imaging-based assessment in reconstructive settings [14].

Despite lipofilling’s extensive clinical usage, no imaging-based data exist to describe the structural regeneration that AFG causes in human tissue exposed to radiation. Ultrasound is still the most practical, affordable, and real-time technique for assessing tissue echogenicity, fat integration, and hypodermal thickness, even if MRI and elastography provide encouraging volumetric and biomechanical data [15].

The aim of this prospective pilot study was to describe ultrasound-detectable structural changes in irradiated postmastectomy chest wall tissues following autologous fat grafting.

## 2. Materials and Methods

### 2.1. Study Design and Patient Selection

This was a prospective single-center pilot study conducted between October 2023 and June 2024, evaluating ultrasound-detectable changes following autologous fat grafting in irradiated chest wall tissues postmastectomy. The study followed a prospective, interventional observational design and was conducted under standardized clinical and imaging protocols. Five female patients were recruited from the Department of Plastic and Reconstructive Surgery at “Victor Babeș” University of Medicine and Pharmacy, Timișoara, Romania.

All patients completed the planned follow-up protocol, and no patient was lost to follow-up. Clinical checks took place at 2 weeks and 1 month, but these were not included in the imaging analysis.

Inclusion criteria

Inclusion criteria were female sex, age ≥18 years, prior radical mastectomy followed by adjuvant external beam chest wall radiotherapy, completion of radiotherapy at least 6 months before enrollment, absence of local recurrence or metastatic disease, presence of clinical or structural signs of radiation-induced soft tissue damage such as subcutaneous atrophy or fibrotic scarring, and indication for autologous fat grafting as part of a reconstructive strategy.

Exclusion criteria

Exclusion criteria included uncontrolled systemic illness such as diabetes or cardiovascular disease, active infection or inflammatory skin disease at the donor or recipient site, prior lipofilling in the same anatomical area, and anticoagulant therapy contraindicating fat grafting.

### 2.2. Anesthesia and Perioperative Care

All procedures were performed under general anesthesia using intravenous sedation. Perioperative antibiotic prophylaxis was administered to all patients according to local hospital protocol. Postoperative thromboprophylaxis consisted of subcutaneous enoxaparin (Clexane^®^) 0.4 mL daily for 5 consecutive days, administered according to institutional standard prophylactic protocols for low-risk elective reconstructive procedures. Patients were encouraged to mobilize on the same day after surgery as part of the standard thromboprophylaxis strategy. No thromboembolic events were recorded during the follow-up period.

### 2.3. Fat Harvesting and Processing

Donor areas included the lower abdomen, flanks, or medial thighs, selected based on adipose tissue availability and patient preference. Klein’s super-wet infiltration technique was employed, with tumescent solution injected and allowed to act for approximately 20 min prior to aspiration. Fat was harvested using a 4-mm blunt-tip cannula connected to a vacuum-assisted aspiration system with a sterile collection canister, which enabled the collection of lipoaspirate under continuous, controlled moderate negative pressure [16].

The aspirated fat was manually washed in a sterile container using five sequential rinses with 250 mL of sterile 0.9% saline solution. After each rinse, the lipoaspirate was allowed to decant for approximately 2 min. The middle purified layer was carefully collected for grafting, while the oil supernatant and erythrocyte-rich infranatant were discarded [17].

This technique was selected for procedural standardization, and the study was not designed to compare harvest efficiency or graft quality with other infiltration approaches.

### 2.4. Fat Injection Technique

Fat grafting was performed using a Coleman-type technique. Multiple small aliquots were injected in a retrograde fashion into multiple planes (superficial subcutaneous, deep subcutaneous, and beneath the mastectomy scar) using 50-mL Luer-lock syringes. Care was taken to avoid overcorrection and ensure uniform distribution. The average grafted volume per patient ranged between 130 and 170 mL per breast, depending on tissue capacity and clinical objectives [18].

No cell-assisted lipotransfer, ADSC isolation, or stromal vascular fraction enrichment was performed. This study used standard autologous fat grafting only.

### 2.5. Ultrasound Evaluation Protocol

High-resolution ultrasound imaging was performed by the same trained operator using a linear high-frequency transducer (12 MHz; GE Healthcare, Logiq series). Patients were examined in a standardized supine position with both arms relaxed alongside the body. Ultrasound was conducted both preoperatively and 3–5 months postoperatively using identical machine settings (frequency, gain, depth) and anatomical landmarks to maximize intra-patient comparability.

The following parameters were assessed:

- Dermal and hypodermal thickness (measured in millimeters);
- Echogenicity (hyperechoic/hypoechoic; homogeneous/heterogeneous);
- Visibility and continuity of fascial planes;
- Presence of post-radiation fibrosis signs (loss of architecture, fibrotic bands);
- Detection of oil cysts, necrosis, or fluid collections.

Still images were stored for each examination in DICOM format. No Doppler mode or elastographic assessment was performed due to equipment limitations and protocol standardization. All measurements were manually performed by the same examiner to reduce variability.

Follow-up evaluations were conducted preoperatively and once postoperatively at 3–5 months. No interim ultrasound assessments were performed.

Image post-processing was limited to the removal of non-diagnostic shadow artifacts. No diagnostic-relevant image features were altered.

All measurements were performed by a single experienced operator; intra- and inter-observer repeatability and measurement error were not formally assessed in this pilot study.

### 2.6. Data Collection and Analysis

Quantitative data were extracted for each patient, with a focus on hypodermal thickness changes (expressed in millimeters and percent change from baseline). Qualitative assessments of echogenicity, fascial delineation, and tissue architecture were recorded by the operator. Given the small sample size and exploratory nature of the study, statistical analysis was limited to descriptive measures.

#### Statistical Analysis

Continuous variables were summarized as mean ± standard deviation and as median with interquartile range (IQR).

Absolute and percentage changes were additionally reported for each patient.

Statistical analysis was performed for descriptive and exploratory purposes. Given the pilot nature of the study and the small sample size, no confirmatory inferential conclusions were intended. Statistical analysis was limited to descriptive measures.

### 2.7. Ethical Considerations

All procedures complied with the ethical principles of the Declaration of Helsinki. Patients were fully informed regarding the nature, objectives, risks, and potential benefits of the study. Written informed consent was obtained prior to inclusion. Study approval was granted by the Institutional Ethics Committee of “Victor Babeș” University of Medicine and Pharmacy, Timișoara (Approval No. 54/25 November 2022).

## 3. Results

All five patients completed the ultrasound follow-up protocol. Hypodermal thickness increased in all cases at 3 to 5 months after lipofilling. Individual values are presented in Table 1. No seromas, oil cysts, or necrotic areas were identified at follow-up.

**Figure 1:**
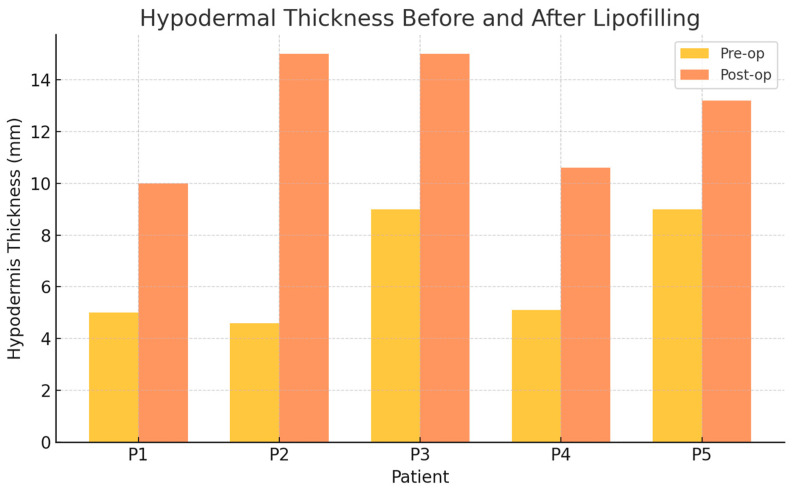
Hypodermal thickness before and after lipofilling in irradiated chest wall tissues. All patients demonstrated measurable postoperative increase in hypodermal thickness in the hypodermis, compatible with adipose integration.

**Figure 2:**
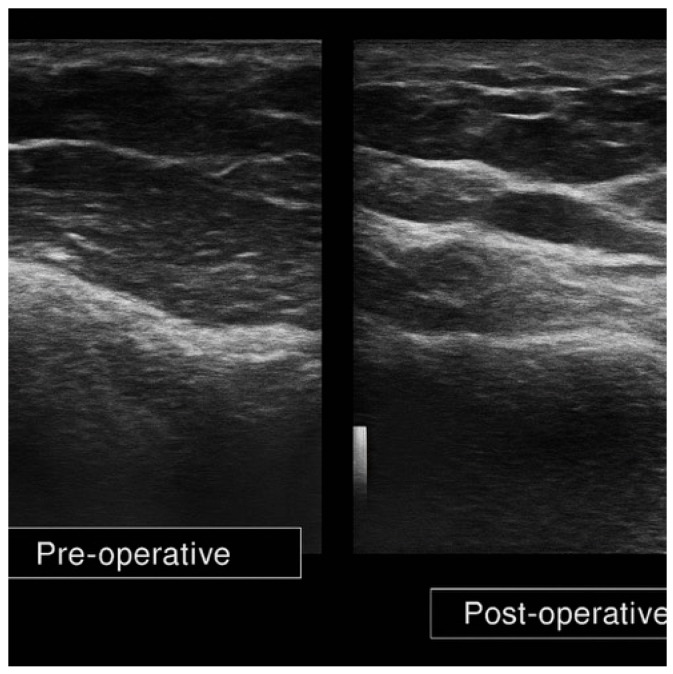
Paired pre- and postoperative ultrasound images (Patient 1) illustrating hypodermal remodeling following autologous fat grafting. Postoperative scan shows increased thickness (from 5.0 mm to 10.0 mm), heterogeneous hypoechoic echotexture, and partial fascial redefinition. No signs of complications.

The preoperative ultrasound pattern was compact and hyperechoic in all patients. Postoperative examinations showed a heterogeneous hypoechoic appearance consistent with structural remodeling. Fascial planes were more clearly delineated in four out of the five patients. Representative preoperative and postoperative images are shown in Figure 1 and Figure 2.

### Descriptive Ultrasound Findings

Preoperative hypodermal thickness ranged from 4.6 to 9.0 mm, with a median of 5.1 mm (IQR 5.0–9.0). Postoperative thickness ranged from 10.0 to 15.0 mm, with a median of 13.2 mm (IQR 10.6–15.0). The median absolute increase was 5.5 mm (IQR 5.0–6.0), corresponding to a mean gain of 6.22 mm. Given the small sample size, the median increase was comparable to the mean value, suggesting limited dispersion among individual measurements.

All patients demonstrated a uniform increase in hypodermal thickness at follow-up.

Preoperative thickness was 6.54 ± 2.14 mm; postoperative thickness was 12.76 ± 2.16 mm.

No postoperative complications were recorded in the study group.

## 4. Discussion

### 4.1. Hypodermal Thickness

In all cases, a measurable increase in hypodermal thickness was observed following lipofilling. Preoperatively, the hypodermis exhibited substantial atrophy, with mean values ranging from 4.6 to 9.0 mm. Postoperative thickness increased consistently in all patients, with final values ranging between 10.0 and 15.0 mm. The mean percentage increase in hypodermal thickness was +110%, with the most pronounced gain observed in Patient 2 (+226%) and the lowest in Patient 5 (+47%). These findings are consistent with the measurable integration of fat grafts into the irradiated subcutaneous plane (Table 1) (Figure 1).

### 4.2. Echogenicity and Tissue Architecture

Preoperative scans demonstrated a compact, homogeneously hyperechogenic hypodermal layer in all patients, suggestive of post-radiation fibrosis and loss of adipose integrity. No clear differentiation between fascial layers was observed, and the acoustic transmission was reduced due to dense connective tissue. Postoperatively, a marked shift to a heterogeneous hypoechogenic pattern was seen in all cases. This architectural change reflects the incorporation of viable adipose tissue, with an echotexture consistent with native subcutaneous fat (Figure 2).

In this study, severe post-radiation fibrosis was primarily suggested by a compact hyperechoic hypodermal pattern with loss of normal layered architecture and the presence of linear fibrotic bands, whereas post-lipofilling ultrasound showed partial restoration of tissue heterogeneity, reduction in fibrotic band visibility, and improved delineation of fascial planes.

### 4.3. Fascial Plane Visibility

Fascial planes within the subcutaneous tissue, poorly defined or absent before surgery, became partially or completely visible postoperatively in 4 out of 5 patients (80%). This reappearance of layered anatomy was interpreted as an indirect sign of extracellular matrix remodeling and reduction in fibrotic density. Patient 1 showed only partial fascial delineation, possibly due to more advanced pre-existing fibrosis or lower fat retention.

### 4.4. Dermal Layer

No significant changes were observed in dermal thickness across the cohort. The dermis remained relatively stable in both thickness and echogenicity, with no evidence of new edema, fibrosis, or disruption. This finding reinforces the subcutaneous localization of the graft and the specificity of regenerative effects within the hypodermal layer.

The stability of dermal thickness and dermal echogenicity throughout follow-up supports the interpretation that the structural changes observed after lipofilling are predominantly confined to the hypodermal layer, consistent with the anatomical level of fat graft placement.

### 4.5. Postoperative Complications and Sonographic Integrity

None of the patients demonstrated sonographic signs of common fat grafting complications such as oil cysts, fat necrosis, seromas, or subcutaneous collections. The grafted regions maintained consistent echogenicity and volume, without evidence of resorption zones or tissue liquefaction at the time of follow-up.

### 4.6. Clinical and Functional Correlation

Although this study did not formally quantify clinical symptoms as in our previous work, all patients reported increased local comfort, improved skin softness, and enhanced scar pliability. These subjective improvements were congruent with the ultrasound findings, particularly the increased hypodermal volume and normalization of echogenicity. In Patient 4, who had an expander in place and underwent DIEP reconstruction after lipofilling, post-graft imaging still showed improved tissue planes and favorable integration, despite the added reconstructive complexity.

These findings are consistent with clinical reconstructive strategies combining fat grafting with regional flaps in irradiated breasts, such as fat-augmented latissimus dorsi flap techniques, which have been shown to improve tissue quality and reconstructive outcomes in previously irradiated fields [19].

### 4.7. Fat Volume and Estimated Retention

The injected fat volume ranged from 120 mL to 250 mL (mean: 164 mL). Based on postoperative ultrasound features, including thickness gain and stable echogenicity, postoperative ultrasound findings suggested stable hypodermal thickness at early follow-up. Patient 5, with the lowest percentage gain in hypodermal thickness, also demonstrated a smaller relative increase in hypodermal thickness compared to other patients, whereas Patient 2 showed the highest increase in hypodermal thickness. These ultrasound-based retention estimations are consistent with previous reports of graft survival in irradiated tissues [11,20].

Postoperative ultrasound analysis in all five patients revealed consistent structural remodeling of the irradiated hypodermis following autologous fat grafting. As an illustrative example, Patient 1 demonstrated a marked increase in hypodermal thickness, from 5.0 mm preoperatively to 10.0 mm postoperatively (+100%), accompanied by a transition from a homogeneously hyperechoic to a heterogeneous hypoechoic echotexture, consistent with imaging features consistent with adipose tissue and reduced fibrotic appearance on ultrasound. Fascial planes, initially absent, became partially redefined, indicating structural reorganization suggested by ultrasound patterns. No postoperative complications such as oil cysts, fat necrosis, or seromas were observed at follow-up (Figure 2).

Table 1 presents a summary of the individual patient data, including interval post-grafting, pre- and postoperative hypodermal measurements, percentage increase, and qualitative changes in echotexture and fascial organization.

This prospective pilot study identified consistent ultrasound patterns suggestive of structural changes in irradiated postmastectomy chest wall tissue, as assessed by high-frequency ultrasound. In all five patients, hypodermal thickness increased substantially following grafting, consistent with imaging features compatible with adipose integration into previously fibrotic subcutaneous tissue [4,8].

Before grafting, ultrasound scans revealed a compact, homogeneously hyperechogenic hypodermal architecture, a hallmark of radiation-induced fibrosis and adipose atrophy [8,10]. Postoperatively, this was replaced by a heterogeneous, hypoechogenic echotexture in all patients, a sonographic feature that correlates with improved fat viability and reduced fibrotic appearance on ultrasound [10,11]. These changes may reflect processes such as extracellular matrix remodeling or neovascularization, but confirmation requires histologic or advanced imaging validation as described in previous experimental studies [12,13,21]. In four out of five cases, previously indistinct fascial planes reappeared following fat grafting—an imaging feature previously described as a surrogate marker of connective tissue normalization [8]. Importantly, no sonographic signs of complications such as oil cysts, fat necrosis, or seromas were observed, suggesting structural stability at early follow-up in irradiated fields [10,13].

Although formal clinical symptom scoring was not conducted in this study, patient-reported improvements in skin pliability and local comfort were consistent with other studies that observed correlation between improved sonographic parameters and subjective recovery following fat grafting in irradiated tissues [10]. These results underscore the value of ultrasound as a noninvasive, real-time tool to monitor regenerative outcomes after lipofilling in the setting of oncologic breast reconstruction [10,11].

High-resolution ultrasound presents a uniquely accessible, safe, and cost-effective modality for evaluating graft behavior and tissue regeneration in breast reconstruction. Compared to magnetic resonance imaging (MRI), which remains expensive and less widely available, ultrasound offers real-time visualization of soft tissue planes, graft dispersion, and fascial anatomy. In a clinical context, this enables the repeated monitoring of post-graft evolution without radiation or contrast administration. Matsumoto et al. emphasized that ultrasound is especially useful in following the spatial distribution and survival of grafted fat in reconstructive procedures, particularly in the early remodeling phase after surgery [22]. In the context of irradiated tissue, ultrasound is particularly well-suited for monitoring subtle structural changes that may correlate with biological recovery. As demonstrated in our study, changes such as increased hypodermal thickness, reduced echogenicity, and fascial plane restoration were clearly visible and required no contrast or special equipment. These parameters may serve as surrogate imaging markers for underlying regenerative events—including angiogenesis, ECM remodeling, and adipose viability—that have traditionally required histologic validation [22,23,24].

Although not formally developed in this study, the combined assessment of hypodermal thickness, echogenicity patterns, fascial plane definition, and fibrotic band visibility could conceptually form a composite ultrasound surrogate index reflecting regenerative activity in irradiated tissues; however, such an index would require validation in larger, longitudinal cohorts.

This pilot study is subject to several limitations. The small sample size (*n* = 5) limits statistical significance and generalizability. Although all patients demonstrated improvement in ultrasound features, larger prospective trials are necessary to confirm these trends across broader patient populations and varying reconstructive contexts. Despite a standardized and consistently applied ultrasound protocol, the assessment relied exclusively on grayscale imaging. Advanced modalities such as Doppler, 3D ultrasound, or MRI were not included, and their absence limited our ability to assess vascular remodeling or volumetric fat retention. Studies have shown that combining fat grafting with cell-enrichment or multimodal imaging provides greater insight into long-term graft survival and angiogenic activity [25]. Moreover, the absence of histological validation means that the sonographic improvements we observed—such as hypodermal thickening and fascial redefinition—remain indirect indicators of tissue regeneration. Rigotti et al. previously demonstrated that these features correspond histologically to reduced fibrosis and restored adipose architecture but we did not perform tissue sampling in this cohort. This study also lacked structured symptom scoring. Although patients reported improved comfort and scar pliability, we did not apply validated clinical metrics such as the BREAST-Q or visual analog scales.

The relatively short follow-up duration (mean 4.0 months) may have precluded the detection of delayed fat resorption, long-term complications, or late-phase regenerative remodeling. Measurements were subject to operator-dependent variability. Despite adherence to a standardized examination protocol, probe positioning and gain settings in soft tissue ultrasonography are inherently variable and may affect reproducibility [26]. Intra- and inter-observer repeatability and absolute measurement error for dermal and hypodermal thickness were not formally quantified in this pilot study. No elastographic evaluation was performed, although it may offer valuable insight into radiation-induced stiffness and its reversal post-lipofilling [27].

In addition, no control group was included, such as non-irradiated grafted tissue or irradiated non-grafted tissue. Therefore, direct comparisons regarding the specific effects of autologous fat grafting versus radiation-related tissue evolution cannot be performed.

## 5. Conclusions

Ultrasound-detectable structural alterations in irradiated postmastectomy chest wall tissues after autologous fat grafting are described in this prospective pilot research. Changes in echogenicity, increased hypodermal thickness, and partial fascial plane repair were regularly noted. These results should be regarded as exploratory because of the limited sample size and descriptive approach. Larger trials are needed to prove clinical importance, although ultrasound seems viable for postoperative surveillance.

Postoperative ultrasound evaluation also demonstrated a consistent transition from a compact hyperechoic architecture to heterogeneous hypoechoic patterns, as well as improved delineation of fascial planes, without sonographic evidence of complications such as oil cysts or fat necrosis.

## Figures and Tables

**Table 1:** Ultrasound characteristics of irradiated chest wall before and after autologous fat grafting.

Patient	Interval (Months)	Hypodermis Thickness (Pre, mm)	Hypodermis Thickness (Post, mm)	Δ Thickness (%)	Echogenicity Change	Fascial Plane (Post-op)	Volume Injected (mL)
1	4	5.0	10.0	+100%	Homogeneous hyperechoic → Heterogeneous hypoechoic	Partial	120
2	5	4.6	15.0	+226%	Homogeneous hyperechoic → Heterogeneous hypoechoic	Clear	150
3	4	9.0	15.0	+67%	Homogeneous hyperechoic → Heterogeneous hypoechoic	Clear	150
4	4	5.1	10.6	+108%	Homogeneous hyperechoic → Heterogeneous hypoechoic	Clear	150
5	3	9.0	13.2	+47%	Homogeneous hyperechoic → Heterogeneous hypoechoic	Clear	250

## Data Availability

The data presented in this study are available on request from the corresponding author. The data are not publicly available due to privacy and ethical restrictions. In Figure 2, images were captured via screen photography; minor shadow artifacts were digitally processed for removal of non-diagnostic shadow artifacts without altering diagnostic content.

## Abbreviations

The following abbreviations are used in this manuscript:

AFG	Autologous Fat Grafting
ADSCs	Adipose-Derived Stem Cells
CT	Computed Tomography
MRI	Magnetic Resonance Imaging
US	Ultrasound
